# Non-linear association between serum folate concentrations and dyslipidemia: Korea National Health and Nutrition Examination Survey 2016-2018

**DOI:** 10.4178/epih.e2022046

**Published:** 2022-05-15

**Authors:** Taiyue Jin, Eun Young Park, Byungmi Kim, Jin-Kyoung Oh

**Affiliations:** 1 Division of Cancer Prevention, National Cancer Control Institute, National Cancer Center, Goyang, Korea; 2 Department of Cancer Control and Population Health, Graduate School of Cancer Science and Policy, National Cancer Center, Goyang, Korea

**Keywords:** Folate, Dyslipidemia, Cholesterol, Triglycerides, Lipoproteins

## Abstract

**OBJECTIVES:**

We aimed to evaluate the association between serum folate concentrations and the prevalence of dyslipidemia.

**METHODS:**

A total of 4,477 adults (2,019 male and 2,458 female) enrolled in the Korea National Health and Nutrition Examination Survey (KNHANES) 2016-2018 were included. Serum samples were used to assess folate concentrations and total cholesterol (TC), triglyceride (TG), low-density lipoprotein (LDL)-cholesterol, and high-density lipoprotein (HDL)-cholesterol levels. Multivariate logistic regression with sampling weights was used to calculate odds ratios (ORs) and 95% confidence intervals (CIs).

**RESULTS:**

Elevated TC, TG, LDL-cholesterol and HDL-cholesterol levels were observed in 506 (11.3%), 646 (14.4%), 434 (9.7%), and 767 (17.1%) participants, respectively. We found non-linear trends between serum folate concentrations and the prevalence of hypercholesterolemia and hyper-LDL cholesterolemia from the restricted cubic smoothing spline. A higher prevalence of hypercholesterolemia was observed among participants in the first tertile of serum folate concentrations (OR,1.38; 95% CI, 1.05 to 1.79) than among those in the second tertile. However, a higher prevalence of hyper-LDL cholesterolemia was identified for both the first and third serum folate concentration tertiles (OR, 1.49; 95% CI, 1.08 to 2.05 and OR, 1.63; 95% CI, 1.20 to 2.20, respectively); furthermore, in these tertiles, the prevalence of hyper-LDL cholesterolemia was more pronounced among obese participants.

**CONCLUSIONS:**

Non-linear associations may exist between serum folate concentrations and the prevalence of hypercholesterolemia and hyper-LDL cholesterolemia in adults. The findings suggest that more accurate recommendations about folate intake and folic acid fortification and supplementation should be provided.

## GRAPHICAL ABSTRACT

[Fig f3-epih-44-e2022046]

## INTRODUCTION

Following the United Nations (UN) High-Level Meeting on the prevention and control of non-communicable diseases (NCDs) [1], the UN General Assembly adopted the goal to “by 2030, reduce by one third premature mortality from NCDs through prevention and treatment and promote mental health and well-being” [2]. Most NCD deaths are attributed to cardiovascular diseases (CVDs), including myocardial infarction (MI), ischemic heart disease, and stroke [3]. In 2019, around 17.9 million deaths worldwide were caused by CVDs, according to the World Health Organization (WHO) [4].

Dyslipidemia is a well-known risk factor for CVDs. According to the 2019 WHO CVD risk charts, the 10-year risk of MI or coronary heart disease (CHD) is significantly increased in both male and female, by 26% and 23% per 1 mmol/L of the total cholesterol (TC) level, respectively [5]. Data from the WHO Global Health Observatory Data Repository showed that in 2008, the prevalence of hypercholesterolemia was highest in Europe (53.7%) and the Americas (47.7%), closely followed by the Western Pacific (36.7%) and South-East Asia (30.3%) [6]. In Korea particularly, from 2005 to 2018, the age-standardized prevalence of hypercholesterolemia increased from 8.0% to 21.4% among adults aged 30 years and over [7].

Folate, an essential water-soluble nutrient belonging to the vitamin B family, is naturally present in a variety of foods such as green leafy vegetables, fruits, nuts, and beans [8]. Folic acid is the oxidized form of folate and is usually found in fortified foods and supplements. According to the Dietary Reference Intakes published by the Institute of Medicine, the recommended dietary allowance for folic acid fortification and supplementation among both adult male and female in the United States is 400 μg/day of dietary folate equivalents [9], which is the same as that for Korean adults [10].

Previous studies have shown an inconsistent association between folate status and risk of CVDs [11-15]. A United States cohort study suggested that low serum folate concentrations were associated with increased CVD mortality [14]. However, several epidemiological studies and meta-analyses have reported that the risk of CVD incidence and mortality was not related to serum folate concentrations [12,13,15]. In relation to CHD, a United States cohort study reported that low serum folate concentrations were significantly associated with decreased risk of CHD in participants aged 55-77 years, but was associated with increased CHD risk in those aged 35-54 years [11]. Furthermore, only a few studies have evaluated whether cholesterol levels are modified by folate status; they have demonstrated inverse associations with levels of TC [16], triglyceride (TG) [16,17], and low-density lipoprotein (LDL)-cholesterol [18], and a positive association with high-density lipoprotein (HDL)-cholesterol levels [17,18].

Given the unclear evidence, it is meaningful to evaluate the association between folate status and risk of dyslipidemia in the general population. In this cross-sectional study, we address the question of whether serum folate concentrations are associated with the prevalence of dyslipidemia using nationally representative data from the Korea National Health and Nutrition Examination Survey (KNHANES).

## MATERIALS AND METHODS

### Study participants

Participants in this study were recruited from the KNHANES, which is a complex, stratified, multi-stage probability sample survey conducted by the Korea Disease Control and Prevention Agency (KDCA). Annually, socio-demographic characteristics, lifestyle factors, anthropometric indices, biochemical profiles, nutritional status, and disease history are collected. For measurement of serum vitamins A, E, and folate, the participants were randomly extracted by survey district, sex, and age (in units of 5 years) from the whole survey population. Further information on the KNHANES can be found elsewhere [19].

**Figure 1.**
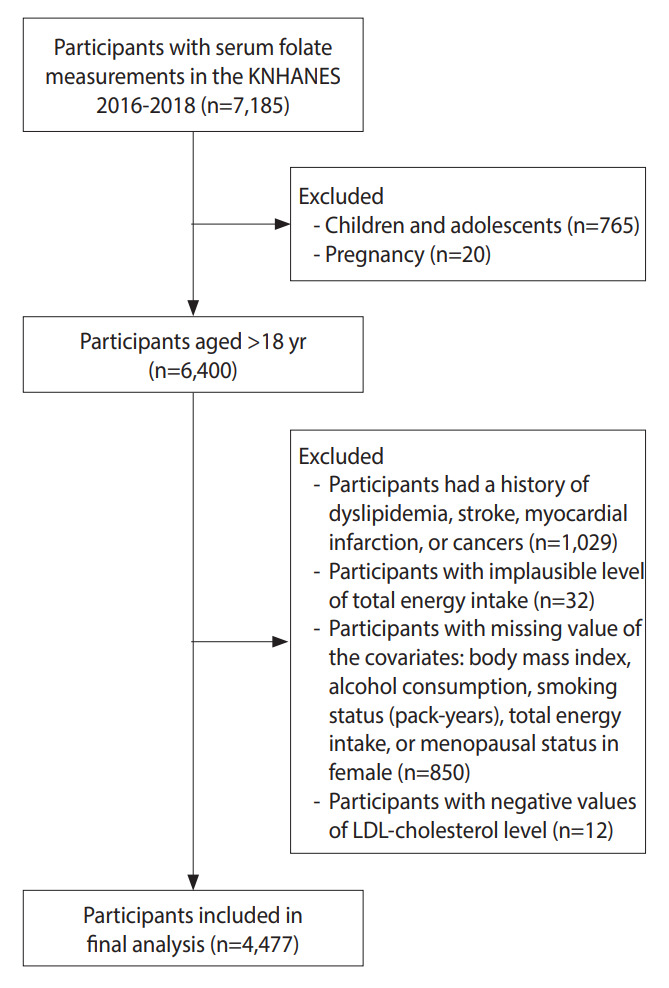
Flow diagram of the inclusion of study participants. KNHANES, Korea National Health and Nutrition Exam ination Survey; LDL, low-density lipoprotein.

A total of 7,185 participants (3,288 male and 3,897 female) with serum folate measurements in the KNHANES 2016-2018 were included. Participants under 19 years of age (n=765) and pregnant female (n=20) were excluded. Participants were also excluded based on the following criteria: having been diagnosed by physicians or treated with medications for dyslipidemia (n=717), CVDs (stroke and MI) (n=120), or cancers (n=192); having an implausible level of total energy intake (± 3 standard deviations from the natural log-transformed mean) (n=32); and not providing information on body mass index (BMI) (n=7), alcohol consumption (n=43), smoking status (n=4), total energy intake (n=710), or menopausal status (n=86). Participants with a negative LDL-cholesterol value were also excluded (n=12). As a result, a total of 4,477 adults (2,019 male and 2,458 female) aged 19 years to 80 years were included in the final analysis. Figure 1 presents a flow diagram of the inclusion of study participants.

### Laboratory analysis

Participants were asked to fast overnight before the blood draw. Serum folate concentration (ng/mL) was measured by the Chemiluminescent Microparticle Immunoassay (CIMA) method using Architect i4000SR (Abbott, Chicago, IL, USA). Serum TC (mg/dL) and TG (mg/dL) levels were determined by the enzymatic methods using the Hitachi Automatic Analyzer 7600-210 (Hitachi, Tokyo, Japan). The homogeneous enzymatic colorimetric method was used to assess the serum HDL-cholesterol level (mg/dL). Information on the laboratory data quality control program can be found elsewhere [20].

### Ascertainment of cases

Serum LDL-cholesterol level (mg/dL) was estimated using the Friedewald formula [21]. In accordance with the Third Report of the Expert Panel on Detection, Evaluation, and Treatment of High Blood Cholesterol in Adults (ATP III) by the National Cholesterol Education Program (NCEP) [22], dyslipidemia was defined as any of the following: (1) hypercholesterolemia with a TC level ≥ 240 mg/dL (6.22 mmol/L); (2) hypertriglyceridemia with a TG level ≥ 200 mg/dL (2.26 mmol/L); (3) hyper-LDL cholesterolemia with an LDL-cholesterol level ≥ 160 mg/dL (4.14 mmol/L); or (4) hypo-HDL cholesterolemia with an HDL-cholesterol level < 40 mg/dL (1.03 mmol/L).

### Covariates

Body weight (kg) and height (m) were measured using a Giant-150N calibrated balance-beam scale (Hana, Seoul, Korea) and a portable stadiometer (Seriter, Bismarck, ND, USA), respectively. BMI (kg/m^2^) was defined as body weight divided by height squared. According to the WHO Western Pacific guideline, a BMI higher than 25 kg/m^2^ was defined as indicating obesity [23]. For past and current smokers, pack-years were calculated by multiplying the duration of smoking (year) by the number of cigarettes (packs/day). The frequency and average amount of any type of alcoholic beverage consumed were asked and used to calculate daily alcohol consumption (drinks/day). Type 2 diabetes and hypertension were defined based on self-reported diagnosis by physicians or treatment. The 24-hour dietary recall method was used to assess total energy intake (kcal/day) and dietary fiber intake (g/day).

### Statistical analysis

Due to the skewed distribution, serum folate concentrations were natural log-transformed. The geometric means (GMs) and 95% confidence intervals (CIs) of serum folate concentrations were calculated according to sex.

We examined whether non-linear associations existed between serum folate concentrations and dyslipidemia. A restricted cubic smoothing spline was constructed with five knots (10th, 25th, 50th, 75th, and 90th percentiles of natural log-transformed serum folate concentrations) using the R package “rms” (https://CRAN.R-project.org/package=rms). PROC SURVEYLOGISTIC was used to calculate the odds ratios (ORs) and 95% CIs. Serum folate concentrations were grouped into tertiles, and the second tertile was regarded as the reference group because participants with mid-range serum folate concentrations appeared to be minimally affected by dyslipidemia in the restricted cubic smoothing spline. The median concentration of serum folate was assigned to each group and used to test the linear trend. Moreover, we also conducted a subgroup analysis according to obesity status (< 25 kg/m^2^ or ≥ 25 kg/m^2^) by comparing the models with or without an interaction term using the likelihood ratio test. In the multivariable analyses, we adjusted for age (years, continuous), sex (for male and female combined), BMI (kg/m^2^, continuous), survey year (2016, 2017, or 2018), smoking status (pack-years, continuous), alcohol consumption (non-drinkers, < 1, 1, > 1 to 2, > 2 to 3, or > 3 drinks/day), menopausal status (for female, premenopausal or postmenopausal), type 2 diabetes (yes or no), hypertension (yes or no), and total energy intake (kcal/day, continuous). In addition, we also adjusted for dietary fiber intake (g/day, continuous) since the fermentation of dietary fiber may promote the microbial biosynthesis of folate by stimulating bacterial growth [24,25]. All analyses except the subgroup analysis were performed with sampling weights due to statistical intricacies. SAS version 9.4 (SAS Institute Inc., Cary, NC, USA) was used for all statistical analyses, and a p-value < 0.05 in 2-sided tests was defined as a significant difference.

### Ethics statement

The study protocol was approved by the Institutional Review Board (IRB) of the KDCA (IRB No. 2018-01-03-PA). Informed consent was confirmed by the IRB.

## RESULTS

### Baseline characteristics

Of the 4,477 participants, 506 (11.3%) had hypercholesterolemia, 646 (14.4%) had hypertriglyceridemia, 434 (9.7%) had hyper-LDL cholesterolemia, and 767 (17.1%) had hypo-HDL cholesterolemia. Participants with any type of dyslipidemia were older and had a higher BMI than those without dyslipidemia (Table 1). Female were more likely to have hypercholesterolemia and hyper-LDL cholesterolemia, whereas male were more likely to have hypertriglyceridemia and hypo-HDL cholesterolemia. All types of dyslipidemia had higher proportions of postmenopausal female than the corresponding group with findings in the acceptable range. Smokers and alcohol drinkers were more likely to have hypertriglyceridemia than their counterparts. Participants who were classified as having hypertriglyceridemia and hypo-HDL cholesterolemia had a higher total energy intake and a higher dietary fiber intake.

### Distribution of serum folate concentrations

The overall GM of serum folate concentrations was 6.35 ng/mL (95% CI, 6.23 to 6.47) (Table 2). Female had higher serum folate concentrations than male (male: GM, 5.41 ng/mL; 95% CI, 5.27 to 5.55; female: GM, 7.47 ng/mL; 95% CI, 7.31 to 7.63).

### Association between serum folate concentrations and the prevalence of dyslipidemia

**Figure 2.**
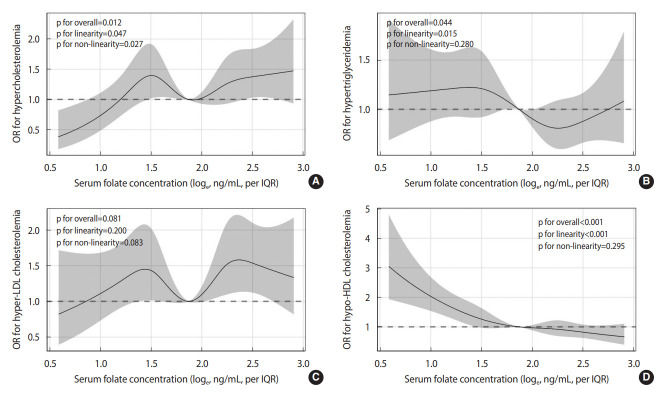
Restricted cubic smoothing splines for serum folate concentrations and the prevalence of dyslipidemia. Restricted cubic smoothing spline models were applied to evaluate the associations between serum folate concentrations and the prevalence of hypercholesterolemia (A), hypertriglyceridemia (B), hyper-LDL cholesterolemia (C), and hypo-HDL cholesterolemia (D), adjusted for age (years, continuous), sex (for male and female combined), body mass index (kg/m^2^, continuous), survey year (2016, 2017, or 2018), smoking status (pack-year, continuous), alcohol consumption (non-drinkers, <1, 1, >1 to 2, >2 to 3, or >3 drinks/day), menopausal status (for female, premenopausal or postmenopausal), type 2 diabetes (yes or no), hypertension (yes or no), and total energy intake (kcal/day, continuous). Participants with the median value of natural log-transformed serum folate concentrations were regarded as the reference group. The solid line represents odds ratio (OR), and the shaded area represents 95% confidence interval. LDL, low-density lipoprotein; HDL, high-density lipoprotein; IQR, interquartile range.

With increasing serum folate concentrations, the OR for hypercholesterolemia showed a significant non-linearity (p for non-linearity= 0.027) (Figure 2A). Notably, although not statistically significant, there were tendencies for non-linear relationships between serum folate concentrations and the prevalence of hypertriglyceridemia and hyper-LDL cholesterolemia (p for non-linearity: 0.280 and 0.083, respectively) (Figure 2B and C). However, in contrast, the prevalence of hypo-HDL cholesterolemia linearly decreased with increments in serum folate concentrations (p for linearity < 0.001) (Figure 2D).

When categorical models were applied, compared with participants in the second tertile, those in the third tertile had a higher prevalence of hypercholesterolemia (OR, 1.38; 95% CI, 1.05 to 1.79) (Table 3). This positive association was persistent only in female (third vs. second tertile: OR, 1.50; 95% CI, 1.07 to 2.09). However, in all the models, no significant linear trend was observed (p for trend: 0.228 for male and female combined, 0.761 for male, and 0.091 for female). After adjusting for potential confounding factors, the prevalence of hypertriglyceridemia was not associated with serum folate concentrations. For hyper-LDL cholesterolemia, a significant association with serum folate concentrations was observed in both the first and the third tertiles (OR, 1.49; 95% CI, 1.08 to 2.05 and OR, 1.63; 95% CI, 1.20 to 2.20, respectively), although the linear trend was not significant (p for trend=0.448). Additionally, female in the third tertile had an 83% higher prevalence of hyper-LDL cholesterolemia (95% CI, 1.27 to 2.65; p for trend=0.190). On the other hand, the prevalence of hypo-HDL cholesterolemia was inversely associated with serum folate concentrations among the first tertile participants compared to those in the second tertile (OR, 1.31; 95% CI, 1.02 to 1.69). Notably, there were significant linear trends in the association between serum folate concentrations and the prevalence of hypo-HDL cholesterolemia (p for trend < 0.001 for male and female combined, 0.005 for male, and 0.006 for female). When we further adjusted for dietary fiber intake, similar associations between serum folate concentrations and the prevalence of dyslipidemia were observed (Supplementary Material 1).

### Subgroup analysis according to obesity status

As shown in Supplementary Material 2, associations between serum folate concentrations and the prevalence of hypercholesterolemia, hypertriglyceridemia, and hypo-HDL cholesterolemia were not modified by obesity status. However, the prevalence of hyper-LDL cholesterolemia was more pronounced among obese participants in both the first and the third tertiles than among those in the second tertile (OR, 1.72; 95% CI, 1.04 to 2.84 and OR, 2.15; 95% CI, 1.28 to 3.63, respectively), although the interaction was not statistically significant (p for interaction=0.702).

## DISCUSSION

In this study, we evaluated the association between serum folate concentrations and the prevalence of dyslipidemia among adults from a nationally representative dataset in Korea. The prevalence of hypercholesterolemia was non-linearly associated with serum folate concentrations, with a higher prevalence among participants with high concentrations of serum folate. Similarly, a non-linear association was found between serum folate concentrations and the prevalence of hyper-LDL cholesterolemia, and the prevalence was significantly higher among participants with low or high serum folate concentrations. In contrast, an inverse association between serum folate concentrations and the prevalence of hypo-HDL cholesterolemia was observed. Given the non-linear associations between serum folate concentrations and cholesterol levels, more accurate recommendations about folate intake and folic acid fortification and supplementation should be provided.

In their 2006 Guidelines on Food Fortification with Micronutrients, the WHO and Food and Agricultural Organization (FAO) of the UN defined serum folate deficiency as less than 4.40 ng/mL (10 nmol/L) [26]. Since 1998, to prevent folate-related diseases, policies for folic acid fortification in food sources have been adopted in several counties. For example, folic acid has been fortified by 154 μg per 100 g of wheat flour in the United States, 150 μg in Canada, and 220 μg in Chile [26]. After fortification, serum folate concentrations increased in all 3 countries: from 5.50 ng/mL to 12.20 ng/mL (median) in the United States [27], from 5.94 ng/mL to 7.96 ng/mL (GM) in Canada [28], and from 4.27 ng/mL to 16.37 ng/mL (mean) in Chile [29]. In contrast, in countries where wheat flour has not been fortified with folic acid, the concentrations of folate are much lower: 7.92 ng/mL for male and 9.33 ng/mL for female (serum mean) in the United Kingdom [30], 6.91 ng/mL (plasma mean) in Spain [31], 7.35 ng/mL (plasma GM) in South China [32], and 8.20 ng/mL for male and 10.90 ng/mL for female (serum mean) in Taiwan [33]. However, folate concentrations in these non-fortified countries, including the Korean population in this study (6.35 ng/mL [serum GM]), are still higher than the cut-off level for folate deficiency suggested in the WHO and FAO guidelines.

Only a few studies have reported the associations between folate status and cholesterol levels. In a German cross-sectional study, LDL-cholesterol levels significantly decreased by 0.164 mmol/L (6.34 mg/dL) per unit increment of plasma folate concentrations, whereas HDL-cholesterol levels increased by 0.094 mmol/L (3.64 mg/dL) [18]. Likewise, in the Chinese population, a positive association between serum folate concentrations and HDL-cholesterol levels has been observed [17]. Additionally, a recent meta-analysis of 34 randomized controlled trials (RCTs) demonstrated that individuals who received folic acid supplementation had significantly lower TC and TG levels (by 3.96 and 9.78 mg/dL, respectively) than those who received placebo [16]. In line with those findings, we identified a linear trend of decreasing hypo-HDL cholesterolemia prevalence with serum folate concentration increments. However, we also observed significant non-linear associations between serum folate concentrations and the prevalence of hypercholesterolemia and hyper-LDL cholesterolemia, with higher prevalence among individuals in the lower or higher serum folate concentration tertiles. The findings of this study need to be replicated in large prospective studies.

The mechanisms of the adverse effects of folate deficiency have been widely investigated. Folate and folic acid in the diet are reduced to tetrahydrofolate (THF) within intestinal cells [34]. THF, which is one of the coenzyme forms of folate, accepts a methyl group from the catabolism of several amino acids (serine, glycine, sarcosine, etc.) [34,35]. The THF derivatives act as one-carbon donors for the remethylation of homocysteine, a well-known independent risk factor for CVDs [13,36]. Methionine generated by homocysteine remethylation is further involved in converting phosphatidylethanolamine (PE) to phosphatidylcholine (PC) [37,38], a major component of biological membranes, bile, and lipoproteins [39]. Folate deficiency reduces PC synthesis by inhibiting the methionine-homocysteine cycle, which further results in hepatic steatosis [40,41]. Additionally, PC synthesis is suppressed because folate deficiency also reduces the activity of the phosphatidylethanolamine N-methyltransferase, a transferase that converts PE to PC [35,39].

We identified a significantly higher prevalence of hypercholesterolemia and hyper-LDL cholesterolemia among participants in the higher serum folate concentration tertile. There is increasing evidence for the adverse health effects of high folate concentrations. Unmetabolized folic acid (UMFA) is an indicator of excessive folate and folic acid intake [42,43] and in a clinical trial [44], UMFA has been found in participants supplied with 200 μg/meal or above of folic acid. Recent studies have also reported that UMFA may lead to the accumulation of dihydrofolate [43,45], the rate-limiting cofactor in converting folic acid to THF [46]. An *in vivo* study has suggested that excessive folic acid intake may also reduce methylenetetrahydrofolate reductase (MTHFR) protein levels in folic acid-supplemented diet-fed mice [42]. Decreased MTHFR levels are also linked to reduced homocysteine remethylation capacity, as MTHFR inhibits the conversion of THF to methyltetrahydrofolate [46,47]. Further prospective studies and RCTs are needed to clarify whether adverse effects of excessive folate and folic acid intake on cholesterol levels exist in the human population.

In this study, the non-linear association between serum folate concentrations and the prevalence of hyper-LDL cholesterolemia was more pronounced among obese participants. An inverse association between serum folate concentrations and BMI has also been reported in previous epidemiological studies [48,49]. However, contrary to our results, a clinical trial found that TC and LDL-cholesterol levels significantly decreased among overweight and obese participants after 3-month supplementation with folic acid [50]. The limited evidence for the effect modification in the association between folate concentrations and the risk of dyslipidemia warrants further investigation.

To our knowledge, we were the first to investigate the non-linear association between folate concentrations and dyslipidemia in the general population. The strengths of this study include the large sample size, adjustment for potential confounding factors, and generalizability. Furthermore, to minimize potential bias, we excluded participants who had been diagnosed with dyslipidemia, CVDs, or cancers before the baseline survey. However, our study had some limitations. Firstly, the findings from this cross-sectional study failed to elucidate a causal link between folate status and risk of dyslipidemia. Secondly, due to the lack of information on folic acid supplement use in the KNHANES, we could not evaluate whether the association between serum folate concentrations and the prevalence of dyslipidemia was modified by dietary folic acid supplementation. We also could not identify participants who supplemented with multivitamins, especially vitamins B_12_, B_6_, and B_2_, which are related to one-carbon metabolism [38]. However, to control for potential dietary confounders [25], we conducted a further analysis that adjusted for dietary fiber intake and found similar associations. Thirdly, despite all efforts to adjust for bias, residual confounding factors may have persisted.

In conclusion, we found that serum folate concentrations were non-linearly associated with hypercholesterolemia and hyper-LDL cholesterolemia, with a higher prevalence among individuals with lower or higher serum folate concentrations. The findings suggest that more accurate recommendations on folate intake and folic acid fortification should be provided, and to prevent dyslipidemia in adults, public health strategies for maintaining adequate folate status should be implemented.

## Figures and Tables

**Figure f3-epih-44-e2022046:**
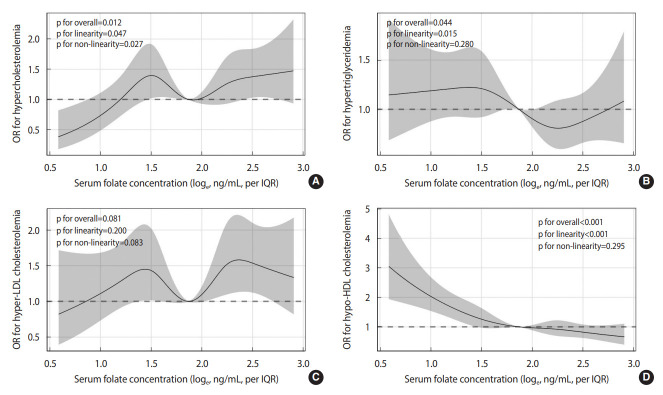


**Table 1.** Baseline characteristics of the study participants according to cholesterol levels

Characteristics		Overall (n=4,477)	TC			TG			LDL-cholesterol			HDL-cholesterol		
			Acceptable (n=3,971)	High (n=506)	p-value	Acceptable (n=3,831)	High (n=646)	p-value	Acceptable (n=4,043)	High (n=434)	p-value	Acceptable (n=3,710)	Low (n=767)	p-value
Age (yr)		44.8±0.3	44.2±0.3	50.1±0.7	<0.001	44.3±0.3	48.1±0.6	<0.001	44.1±0.3	51.8±0.8	<0.001	44.0±0.3	48.7±0.7	<0.001
Sex														
	Male	2,019 (45.1)	1,808 (45.5)	211 (41.7)	0.003	1,590 (41.5)	429 (66.4)	<0.001	1,847 (45.7)	172 (39.6)	<0.001	1,489 (40.1)	530 (69.1)	<0.001
	Female	2,458 (54.9)	2,163 (54.5)	295 (58.3)		2,241 (58.5)	217 (33.6)		2,196 (54.3)	262 (60.4)		2,221 (59.9)	237 (30.9)	
BMI (kg/m^2^)		23.7±0.1	23.6±0.1	25.0±0.2	<0.001	23.4±0.1	25.8±0.1	<0.001	23.6±0.1	24.7±0.2	<0.001	23.4±0.1	25.6±0.1	<0.001
	Normal weight	2,030 (45.3)	1,865 (47.0)	165 (32.6)	<0.001	1,902 (49.6)	128 (19.8)	<0.001	1,875 (46.4)	155 (35.7)	0.001	1,855 (50.0)	175 (22.8)	<0.001
	Overweight	994 (22.2)	867 (21.8)	127 (25.1)		845 (22.1)	149 (23.1)		883 (21.8)	111 (25.6)		805 (21.7)	189 (24.6)	
	Obesity	1,453 (32.5)	1,239 (31.2)	214 (42.3)		1,084 (28.3)	369 (57.1)		1,285 (31.8)	168 (38.7)		1,050 (28.3)	403 (52.5)	
Smoking status (pack-year)														
	Never smokers	2,736 (61.1)	2,435 (61.3)	301 (59.5)	0.511	2,492 (65.0)	244 (37.8)	<0.001	2,468 (61.0)	268 (61.8)	0.326	2,415 (65.1)	321 (41.9)	<0.001
	<10	764 (17.1)	680 (17.1)	84 (16.6)		637 (16.6)	127 (19.7)		698 (17.3)	66 (15.2)		608 (16.4)	156 (20.3)	
	10 to <20	389 (8.7)	330 (8.3)	59 (11.7)		286 (7.5)	103 (15.9)		341 (8.4)	48 (11.1)		287 (7.7)	102 (13.3)	
	20 to <30	255 (5.7)	231 (5.8)	24 (4.7)		174 (4.5)	81 (12.5)		232 (5.7)	23 (5.3)		184 (5.0)	71 (9.3)	
	30 to <40	177 (4.0)	154 (3.9)	23 (4.5)		124 (3.2)	53 (8.2)		159 (3.9)	18 (4.1)		114 (3.1)	63 (8.2)	
	≥40	156 (3.5)	141 (3.6)	15 (3.0)		118 (3.1)	38 (5.9)		145 (3.6)	11 (2.5)		102 (2.7)	54 (7.0)	
Alcohol consumption (drink/day)														
	Non-drinkers	1,029 (23.0)	899 (22.6)	130 (25.7)	0.440	890 (23.2)	139 (21.5)	<0.001	905 (22.4)	124 (28.6)	0.001	803 (21.6)	226 (29.5)	0.101
	<1	2,363 (52.8)	2,100 (52.9)	263 (52.0)		2,105 (54.9)	258 (39.9)		2,121 (52.5)	242 (55.8)		1,981 (53.4)	382 (49.8)	
	1	161 (3.6)	143 (3.6)	18 (3.6)		135 (3.5)	26 (4.0)		149 (3.7)	12 (2.8)		134 (3.6)	27 (3.5)	
	>1 to 2	325 (7.3)	297 (7.5)	28 (5.5)		268 (7.0)	57 (8.8)		304 (7.5)	21 (4.8)		288 (7.8)	37 (4.8)	
	>2 to 3	209 (4.7)	186 (4.7)	23 (4.5)		163 (4.3)	46 (7.1)		197 (4.9)	12 (2.8)		175 (4.7)	34 (4.4)	
	>3	390 (8.7)	346 (8.7)	44 (8.7)		270 (7.0)	120 (18.6)		367 (9.1)	23 (5.3)		329 (8.9)	61 (8.0)	
Menopausal status (for female)														
	Premenopausal	1,516 (61.7)	1,414 (65.4)	102 (34.6)	<0.001	1,423 (63.5)	93 (42.9)	<0.001	1,429 (65.1)	87 (33.2)	<0.001	1,415 (63.7)	101 (42.6)	<0.001
	Postmenopausal	942 (38.3)	749 (34.6)	193 (65.4)		818 (36.5)	124 (57.1)		767 (34.9)	175 (66.8)		806 (36.3)	136 (57.4)	
Total energy intake (kcal/day)		2,068.6±16.8	2,074.0±17.0	2,023.9±53.9	0.079	2,031.7±17.2	2,294.2±49.4	<0.001	2,082.6±17.1	1,929.7±55.1	<0.001	2,050.3±17.7	2,155.9±42.8	0.005
Survey year														
	2016	2,005 (44.8)	1,781 (44.9)	224 (44.3)	0.432	1,697 (44.3)	308 (47.7)	0.273	1,821 (45.0)	184 (42.4)	0.265	1,645 (44.3)	360 (46.9)	0.217
	2017	1,205 (26.9)	1,063 (26.8)	142 (28.1)		1,033 (27.0)	172 (26.6)		1,086 (26.9)	119 (27.4)		1,015 (27.4)	190 (24.8)	
	2018	1,267 (28.3)	1,127 (28.4)	140 (27.7)		1,101 (28.7)	166 (25.7)		1,136 (28.1)	131 (30.2)		1,050 (28.3)	217 (28.3)	
Type 2 diabetes														
	Yes	240 (5.4)	225 (5.7)	15 (3.0)	0.111	193 (5.0)	47 (7.3)	0.083	229 (5.7)	11 (2.5)	0.059	153 (4.1)	87 (11.3)	<0.001
	No	4,237 (94.6)	3,746 (94.3)	491 (97.0)		3,638 (95.0)	599 (92.7)		3,814 (94.3)	423 (97.5)		3,557 (95.9)	680 (88.7)	
Hypertension														
	Yes	715 (16.0)	635 (16.0)	80 (15.8)	0.590	583 (15.2)	132 (20.4)	0.054	644 (15.9)	71 (16.4)	0.565	531 (14.3)	184 (24.0)	<0.001
	No	3,762 (84.0)	3,336 (84.0)	426 (84.2)		3,248 (84.8)	514 (79.6)		3,399 (84.1)	363 (83.6)		3,179 (85.7)	583 (76.0)	
Dietary fiber intake (g/day)		25.1±0.3	25.0±0.3	26.4±0.8	0.035	24.8±0.3	27.3±0.7	<0.001	25.1±0.3	25.9±0.9	0.352	24.9±0.3	26.3±0.6	0.026
Serum folate concentration (ng/mL)		7.2±0.1	7.1±0.1	7.9±0.2	<0.001	7.2±0.1	6.6±0.2	<0.001	7.1±0.1	8.0±0.2	<0.001	7.3±0.1	6.2±0.1	<0.001

Values are presented as mean±standard error or number (%). TC, total cholesterol; TG, triglyceride; LDL, low-density lipoprotein; HDL, high-density lipoprotein; SE, standard error; BMI, body mass index.

**Table 2.** Distribution of serum folate concentrations (ng/mL) according to sex

Variables	n (%)	GM (95% CI)	Min	10%	25th	Median	75th	90%	Max
Overall	4,477 (100)	6.35 (6.23, 6.47)	1.50	3.26	4.45	6.26	9.09	12.40	35.90
Male	2,019 (45.1)	5.41 (5.27, 5.55)	1.50	2.88	3.71	5.30	7.46	10.39	21.90
Female	2,458 (54.9)	7.47 (7.31, 7.63)	1.50	4.08	5.41	7.43	10.42	13.42	35.90

GM, geometric mean; CI, confidence interval; Min, minimum; Max, maximum.

**Table 3.** Associations between serum folate concentrations and the prevalence of dyslipidemia according to sex

Variables		Concentration range (ng/mL)	Overall			Male			Female		
			Case/total	Crude	Adjusted^1^	Case/total	Crude	Adjusted^1^	Case/total	Crude	Adjusted^1^
Hypercholesterolemia											
	Tertile 1	1.5-5.2	158/1,501	1.04 (0.79, 1.37)	1.14 (0.84, 1.55)	108/967	1.14 (0.77, 1.68)	1.17 (0.77, 1.76)	50/534	0.97 (0.63, 1.51)	1.09 (0.68, 1.74)
	Tertile 2	5.3-8.2	155/1,509	1.00 (reference)	1.00 (reference)	60/642	1.00 (reference)	1.00 (reference)	95/867	1.00 (reference)	1.00 (reference)
	Tertile 3	8.3-35.9	193/1,467	1.57 (1.22, 2.03)	1.38 (1.05, 1.79)	43/410	1.13 (0.72, 1.80)	1.13 (0.71, 1.80)	150/1,057	1.69 (1.22, 2.34)	1.50 (1.07, 2.09)
	p for trend			0.005	0.228		0.846	0.761		0.005	0.091
Hypertriglyceridemia											
	Tertile 1	1.5-5.2	283/1,501	1.28 (1.02, 1.61)	1.05 (0.82, 1.35)	218/967	0.99 (0.74, 1.32)	0.99 (0.73, 1.34)	65/534	1.35 (0.90, 2.02)	1.28 (0.81, 2.02)
	Tertile 2	5.3-8.2	214/1,509	1.00 (reference)	1.00 (reference)	134/642	1.00 (reference)	1.00 (reference)	80/867	1.00 (reference)	1.00 (reference)
	Tertile 3	8.3-35.9	149/1,467	0.74 (0.58, 0.95)	0.85 (0.65, 1.11)	77/410	0.94 (0.66, 1.35)	0.94 (0.65, 1.37)	72/1,057	0.82 (0.56, 1.21)	0.80 (0.53, 1.21)
	p for trend			<0.001	0.178		0.811	0.835		0.037	0.076
Hyper-LDL cholesterolemia											
	Tertile 1	1.5-5.2	135/1,501	1.22 (0.90, 1.64)	1.49 (1.08, 2.05)	85/967	1.26 (0.83, 1.91)	1.34 (0.87, 2.07)	50/534	1.33 (0.85, 2.09)	1.62 (1.00, 2.62)
	Tertile 2	5.3-8.2	119/1,509	1.00 (reference)	1.00 (reference)	48/642	1.00 (reference)	1.00 (reference)	71/867	1.00 (reference)	1.00 (reference)
	Tertile 3	8.3-35.9	180/1,467	1.99 (1.48, 2.68)	1.63 (1.20, 2.20)	39/410	1.37 (0.80, 2.36)	1.26 (0.72, 2.19)	141/1,057	2.13 (1.49, 3.04)	1.83 (1.27, 2.65)
	p for trend			0.002	0.448		0.975	0.613		0.005	0.190
Hypo-HDL cholesterolemia											
	Tertile 1	1.5-5.2	353/1,501	1.54 (1.23, 1.94)	1.31 (1.02, 1.69)	285/967	1.28 (0.96, 1.70)	1.35 (1.00, 1.82)	68/534	1.22 (0.82, 1.83)	1.23 (0.78, 1.93)
	Tertile 2	5.3-8.2	238/1,509	1.00 (reference)	1.00 (reference)	152/642	1.00 (reference)	1.00 (reference)	86/867	1.00 (reference)	1.00 (reference)
	Tertile 3	8.3-35.9	176/1,467	0.71 (0.55, 0.92)	0.77 (0.59, 1.01)	93/410	0.96 (0.68, 1.35)	0.85 (0.59, 1.21)	83/1,057	0.75 (0.50, 1.10)	0.68 (0.45, 1.02)
	p for trend			<0.001	<0.001		0.046	0.005		0.019	0.006

Values are presented as odds ratio (95% confidence interval). LDL, low-density lipoprotein; HDL, high-density lipoprotein.

1 Multivariate logistic regression model adjusted for age (years, continuous), sex (for male and female combined), body mass index (kg/m^2^, continuous), survey year (2016, 2017, or 2018), smoking status (pack-year, continuous), alcohol consumption (non-drinkers, <1, 1, >1 to 2, >2 to 3, or >3 drinks/day), menopausal status (for female, premenopausal or postmenopausal), type 2 diabetes (yes or no), hypertension (yes or no), and total energy intake (kcal/day, continuous).

## SUPPLEMENTARY MATERIALS

Non-linear association between serum folate concentrations and dyslipidemia: Korea National Health and Nutrition Examination Survey 2016-2018

Supplementary Material 1. Associations between serum folate concentrations and the prevalence of dyslipidemia according to sex (further adjusted for dietary fiber intake)

Supplementary Material 2. Associations between serum folate concentrations and the prevalence of dyslipidemia according to obesity status
