# Supplementary material for: Non-linear association between serum folate concentrations and dyslipidemia: Korea National Health and Nutrition Examination Survey 2016-2018
Source: Epidemiol Health. 2022 May 15;44:e2022046. doi: 10.4178/epih.e2022046 (PMC9684009; doi:10.4178/epih.e2022046)
Supplement: Supplementary Material 1. — Associations between serum folate concentrations and the prevalence of dyslipidemia according to sex (further adjusted for dietary fiber intake) [file epih-44-e2022046-suppl1.docx]

**Supplementary Material 1.** Associations between serum folate concentrations and the prevalence of dyslipidemia according to sex (further adjusted for dietary fiber intake)

|  | Concentration range (ng/mL) | Overall | | | Male | | | Female | | |
| --- | --- | --- | --- | --- | --- | --- | --- | --- | --- | --- |
|  |  | Case/total | Crude | Adjusted^1^ | Case/total | Crude | Adjusted^1^ | Case/total | Crude | Adjusted^1^ |
| Hypercholesterolemia | |  |  |  |  |  |  |  |  |  |
| Tertile 1 | 1.5 - 5.2 | 158/1,501 | 1.04 (0.79, 1.37) | 1.15 (0.85, 1.56) | 108/967 | 1.14 (0.77, 1.68) | 1.18 (0.78, 1.77) | 50/534 | 0.97 (0.63, 1.51) | 1.10 (0.68, 1.76) |
| Tertile 2 | 5.3 - 8.2 | 155/1,509 | 1.00 (Reference) | 1.00 (Reference) | 60/642 | 1.00 (Reference) | 1.00 (Reference) | 95/867 | 1.00 (Reference) | 1.00 (Reference) |
| Tertile 3 | 8.3 - 35.9 | 193/1,467 | 1.57 (1.22, 2.03) | 1.37 (1.05, 1.79) | 43/410 | 1.13 (0.72, 1.80) | 1.12 (0.70, 1.80) | 150/1,057 | 1.69 (1.22, 2.34) | 1.49 (1.07, 2.08) |
| p for trend |  |  | 0.005 | 0.256 |  | 0.846 | 0.720 |  | 0.005 | 0.099 |
| Hypertriglyceridemia | |  |  |  |  |  |  |  |  |  |
| Tertile 1 | 1.5 - 5.2 | 283/1,501 | 1.28 (1.02, 1.61) | 1.06 (0.82, 1.37) | 218/967 | 0.99 (0.74, 1.32) | 1.00 (0.74, 1.36) | 65/534 | 1.35 (0.90, 2.02) | 1.30 (0.82, 2.05) |
| Tertile 2 | 5.3 - 8.2 | 214/1,509 | 1.00 (Reference) | 1.00 (Reference) | 134/642 | 1.00 (Reference) | 1.00 (Reference) | 80/867 | 1.00 (Reference) | 1.00 (Reference) |
| Tertile 3 | 8.3 - 35.9 | 149/1,467 | 0.74 (0.58, 0.95) | 0.84 (0.65, 1.10) | 77/410 | 0.94 (0.66, 1.35) | 0.94 (0.65, 1.36) | 72/1,057 | 0.82 (0.56, 1.21) | 0.79 (0.52, 1.19) |
| p for trend |  |  | <.001 | 0.154 |  | 0.811 | 0.771 |  | 0.037 | 0.058 |
| Hyper-LDL cholesterolemia | |  |  |  |  |  |  |  |  |  |
| Tertile 1 | 1.5 - 5.2 | 135/1,501 | 1.22 (0.90, 1.64) | 1.48 (1.07, 2.04) | 85/967 | 1.26 (0.83, 1.91) | 1.31 (0.85, 2.03) | 50/534 | 1.33 (0.85, 2.09) | 1.63 (1.01, 2.62) |
| Tertile 2 | 5.3 - 8.2 | 119/1,509 | 1.00 (Reference) | 1.00 (Reference) | 48/642 | 1.00 (Reference) | 1.00 (Reference) | 71/867 | 1.00 (Reference) | 1.00 (Reference) |
| Tertile 3 | 8.3 - 35.9 | 180/1,467 | 1.99 (1.48, 2.68) | 1.63 (1.21, 2.21) | 39/410 | 1.37 (0.80, 2.36) | 1.27 (0.72, 2.22) | 141/1,057 | 2.13 (1.49, 3.04) | 1.83 (1.26, 2.65) |
| p for trend |  |  | 0.002 | 0.410 |  | 0.975 | 0.701 |  | 0.005 | 0.192 |
| Hypo-HDL cholesterolemia | |  |  |  |  |  |  |  |  |  |
| Tertile 1 | 1.5 - 5.2 | 353/1,501 | 1.54 (1.23, 1.94) | 1.31 (1.01, 1.68) | 285/967 | 1.28 (0.96, 1.70) | 1.32 (0.98, 1.79) | 68/534 | 1.22 (0.82, 1.83) | 1.26 (0.80, 1.99) |
| Tertile 2 | 5.3 - 8.2 | 238/1,509 | 1.00 (Reference) | 1.00 (Reference) | 152/642 | 1.00 (Reference) | 1.00 (Reference) | 86/867 | 1.00 (Reference) | 1.00 (Reference) |
| Tertile 3 | 8.3 - 35.9 | 176/1,467 | 0.71 (0.55, 0.92) | 0.77 (0.59, 1.01) | 93/410 | 0.96 (0.68, 1.35) | 0.86 (0.60, 1.22) | 83/1,057 | 0.75 (0.50, 1.10) | 0.66 (0.44, 1.00) |
| p for trend |  |  | <.001 | <.001 |  | 0.046 | 0.009 |  | 0.019 | 0.004 |

LDL, low-density lipoprotein; HDL, high-density lipoprotein; OR, odds ratio; CI, confidence interval. ^1^ Multivariate logistic regression model adjusted for age (years, continuous), sex (for male and female combined), body mass index (kg/m^2^, continuous), survey year (2016, 2017, and 2018), smoking status (pack-year, continuous), alcohol consumption (non-drinkers, <1 drink/day, 1 drink/day, >1 to 2 drinks/day, >2 to 3 drinks/day, and >3 drinks/day), menopausal status (for female, premenopausal and postmenopausal), type 2 diabetes (yes or no), hypertension (yes or no), total energy intake (kcal/day, continuous), and dietary fiber intake (g/day, continuous)
