# Supplementary material for: Non-linear association between serum folate concentrations and dyslipidemia: Korea National Health and Nutrition Examination Survey 2016-2018
Source: Epidemiol Health. 2022 May 15;44:e2022046. doi: 10.4178/epih.e2022046 (PMC9684009; doi:10.4178/epih.e2022046)
Supplement: Supplementary Material 2. — Associations between serum folate concentrations and the prevalence of dyslipidemia according to obesity status [file epih-44-e2022046-suppl2.docx]

**Supplementary Material 2.** Associations between serum folate concentrations and the prevalence of dyslipidemia according to obesity status ^1^

|  | Non-obesity | | Obesity | | p for interaction |
| --- | --- | --- | --- | --- | --- |
|  | Case/total | OR (95% CI) ^2^ | Case/total | OR (95% CI) ^2^ |  |
| Hypercholesterolemia |  |  |  |  | 0.488 |
| Tertile 1 | 83/933 | 1.27 (0.83, 1.93) | 75/568 | 1.03 (0.68, 1.58) |  |
| Tertile 2 | 88/1,017 | 1.00 (Reference) | 67/492 | 1.00 (Reference) |  |
| Tertile 3 | 121/1,074 | 1.30 (0.90, 1.88) | 72/393 | 1.49 (0.96, 2.31) |  |
| *p* for trend |  | 0.774 |  | 0.135 |  |
| Hypertriglyceridemia |  |  |  |  | 0.882 |
| Tertile 1 | 113/933 | 1.22 (0.82, 1.81) | 170/568 | 0.95 (0.67, 1.33) |  |
| Tertile 2 | 90/1,017 | 1.00 (Reference) | 124/492 | 1.00 (Reference) |  |
| Tertile 3 | 74/1,074 | 0.93 (0.62, 1.40) | 75/393 | 0.79 (0.54, 1.15) |  |
| *p* for trend |  | 0.229 |  | 0.420 |  |
| Hyper-LDL cholesterolemia | |  |  |  | 0.702 |
| Tertile 1 | 73/933 | 1.44 (0.94, 2.19) | 62/568 | 1.72 (1.04, 2.84) |  |
| Tertile 2 | 76/1,017 | 1.00 (Reference) | 43/492 | 1.00 (Reference) |  |
| Tertile 3 | 117/1,074 | 1.40 (0.97, 2.02) | 63/393 | 2.15 (1.28, 3.63) |  |
| *p* for trend |  | 0.885 |  | 0.420 |  |
| Hypo-HDL cholesterolemia | |  |  |  | 0.762 |
| Tertile 1 | 162/933 | 1.64 (1.16, 2.34) | 191/568 | 1.07 (0.74, 1.53) |  |
| Tertile 2 | 110/1,017 | 1.00 (Reference) | 128/492 | 1.00 (Reference) |  |
| Tertile 3 | 92/1,074 | 0.87 (0.60, 1.26) | 84/393 | 0.65 (0.43, 0.98) |  |
| *p* for trend |  | 0.001 |  | 0.015 |  |

LDL, low-density lipoprotein; HDL, high-density lipoprotein; OR, odds ratio; CI, confidence interval. ^1^ Obesity status was defined as a BMI higher than 25 kg/m^2^. ^2^ Multivariate logistic regression model adjusted for age (years, continuous), sex (for male and female combined), body mass index (kg/m^2^, continuous), survey year (2016, 2017, and 2018), smoking status (pack-year, continuous), alcohol consumption (non-drinkers, <1 drink/day, 1 drink/day, >1 to 2 drinks/day, >2 to 3 drinks/day, and >3 drinks/day), menopausal status (for female, premenopausal and postmenopausal), type 2 diabetes (yes or no), hypertension (yes or no), and total energy intake (kcal/day, continuous).
